# A Case Report of Foreign Body Embolization

**DOI:** 10.7759/cureus.4917

**Published:** 2019-06-17

**Authors:** Hemanth Boppana, Shravan Teelucksingh, L K Teja Boppana, Sateesh Sakhamuri

**Affiliations:** 1 Internal Medicine, The University of the West Indies, St. Augustine, TTO; 2 Radiology, The University of the West Indies, St. Augustine, TTO; 3 Internal Medicine, Medical Associates Hospital, St. Joseph, TTO; 4 Internal Medicine - Pulmonology, The University of the West Indies, St. Augustine, TTO

**Keywords:** prostate brachytherapy, brachytherapy embolization, seed migration, prostate cancer

## Abstract

Prostate brachytherapy (BT) seed embolization to the lung is a rare complication, with <1% of all seeds migrating post-implantation. Here, we present the case of a 63-year-old male who presented with a history of chest pain and intermittent dyspnea at rest for the last four months.

## Introduction

Prostate brachytherapy embolization to the lung is rare and is influenced by factors such as planning volume, number of seeds, seed placement, and types of seeds used. A thorough history and clinical examination, followed by radiological investigations, are useful in making the diagnosis.

## Case presentation

A 63-year-old male patient with a past medical history of prostate cancer diagnosed in 2018 presented to our outpatient clinic with a four-month history of central chest pain with left-sided radiation and intermittent dyspnea at rest. The chest pain has persisted since a motor vehicle accident four months ago for which he visited the emergency room and was told that he had a superficial soft tissue injury and required no further follow-up. He denied orthopnea, paroxysmal nocturnal dyspnea, palpitations, cough, fever, weight loss, night sweats, and reported healthy bladder and bowel function. Past medical history was negative for diabetes, hypertension, dyslipidemia and was only significant for prostate cancer for which he received brachytherapy with stranded seeds nine months ago. The patient denied any surgeries, family history of medical conditions, alcohol, or tobacco use. He lives alone, is currently not sexually active, and is a retired prison officer. There is no significant drug history.

On physical exam, he appeared to be in no apparent cardiopulmonary distress. His vital signs were normal, with a blood pressure of 130/80 mmHg, pulse of 72/min, respiratory rate of 16/min, and ambient air oxygen saturation of 99%. Examination of the head and neck showed pink mucous membranes. The cardiovascular, respiratory, and abdominal examinations were normal.

**Figure 1:**
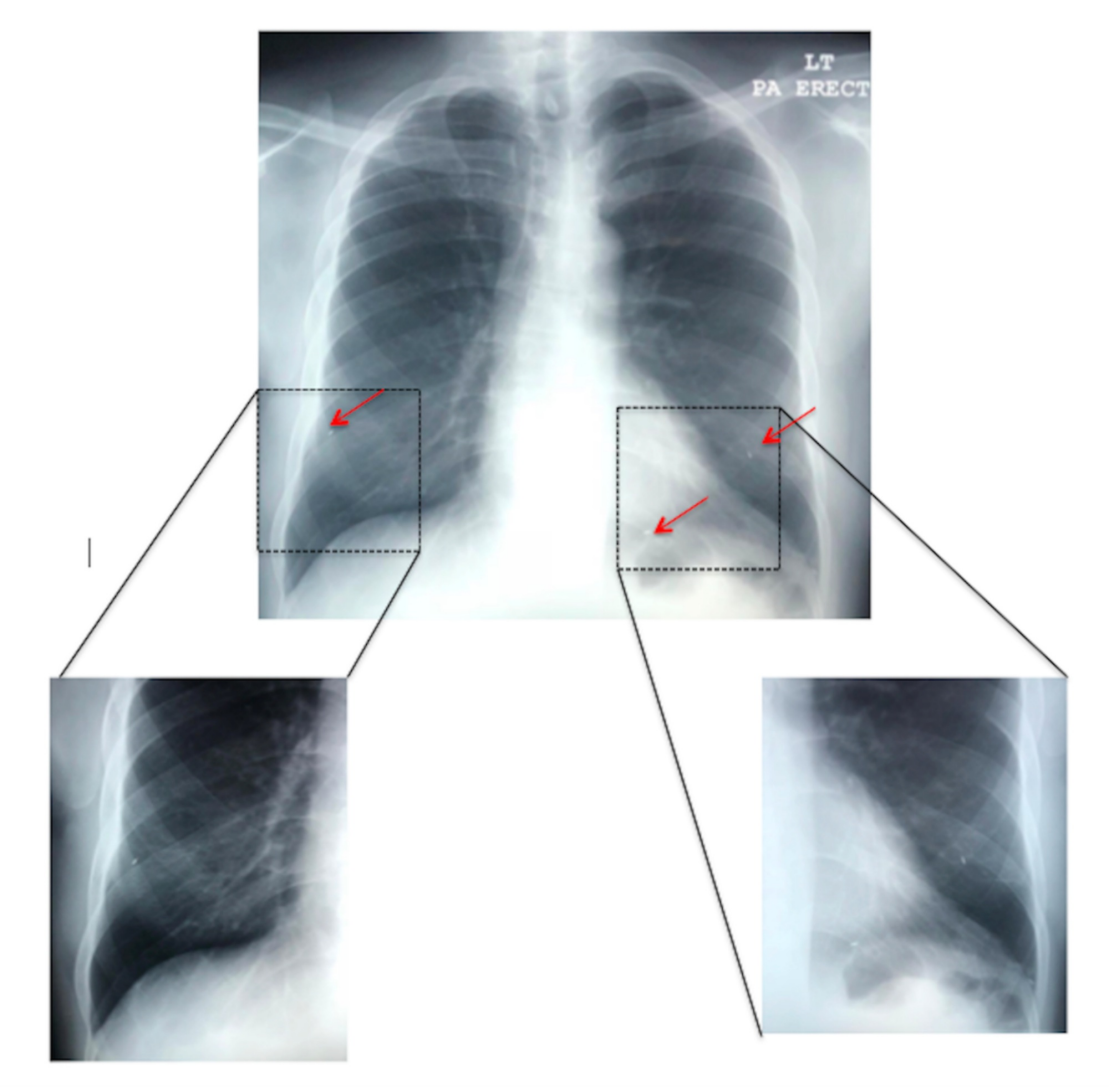
Frontal (posterior to anterior technique) chest radiograph demonstrating three migrated brachytherapy seeds in the lower lung zones bilaterally (red arrows).

**Figure 2:**
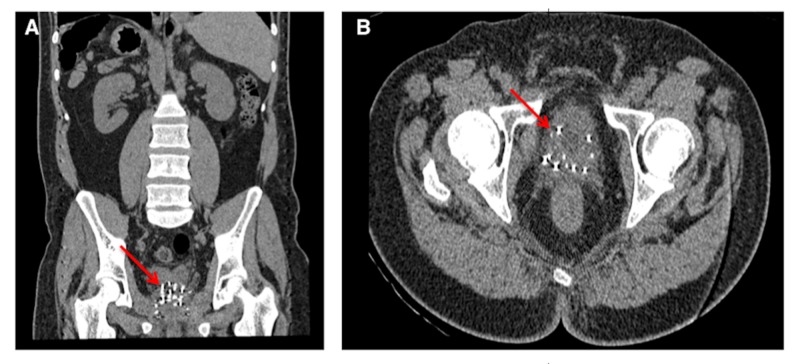
Coronal (A) and axial (B) CT of the abdomen and pelvis without contrast showing multiple 4 mm x 0.8 mm densities within the prostate gland, consistent with brachytherapy seeds.

**Figure 3:**
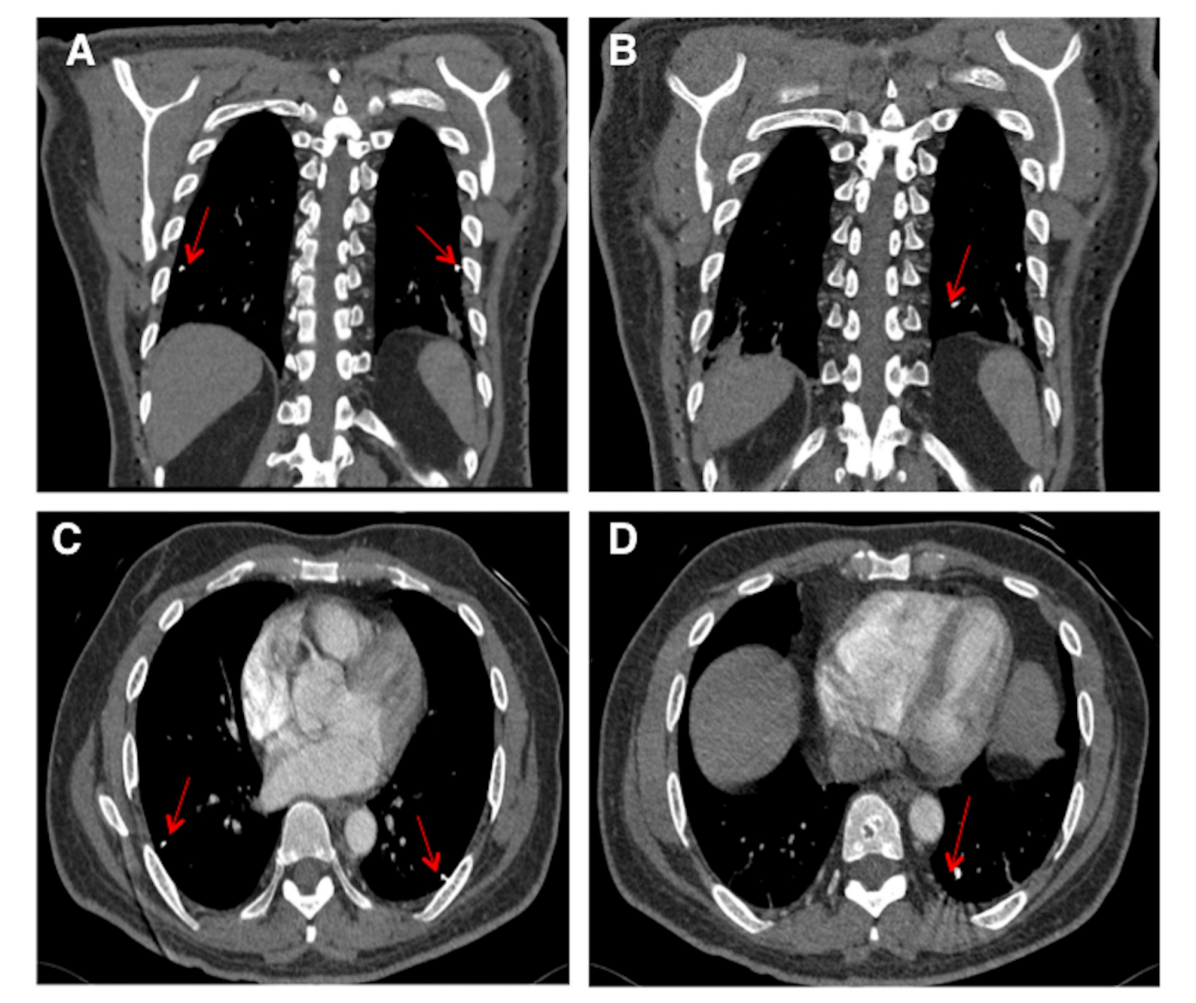
Coronal (A and B) and axial (C and D) CT of the chest with contrast showing the three brachytherapy seeds, which were visualized on chest radiograph.

Laboratory investigations showed that the complete blood count and biochemical investigations, including C-reactive protein and D-Dimer, were within normal limits. An electrocardiogram (EKG) showed normal sinus rhythm. Chest radiograph showed normal heart shadow and three, scattered, metallic-appearing foreign objects (red arrows) in the lower lung zones bilaterally (Figure 1). The EKG was within normal limits, with an ejection fraction (EF) of 60% and preserved systolic function. Non-contrast computed tomography (CT) abdomen and pelvis was done, which showed multiple 4 mm x 0.8 mm linear metallic foreign bodies (red arrows) (Figure 2), followed by a CT chest with contrast (Figure 3), which showed the same 4 mm x 0.8 mm foreign bodies (red arrows) consistent with the objects visualized in the chest radiograph.

A diagnosis of prostate brachytherapy embolization was made, and the patient was reassured. A biopsy of the visualized seeds not done. On a follow-up visit three months later, spirometry was done and was within normal limits. The patient was doing well.

## Discussion

Brachytherapy (BT), as a definition, is derived from Greek words for ‘short distance’ (brachios) and ‘treatment’ (therapy) and refers to the use of encapsulated radio nucleotides within or close to a tumor. BT has been used for close to a century for the treatment of prostate cancer, gynecologic cancers, and head and neck cancers, among others. BT is an internal radiation therapy that is applied either in a permanent manner (called seed implantation) or in a temporary manner, often through the use of catheters into which the radioactive sources are placed. When permanent BT is being employed, the seeds are left inside the body [1].

In our case, BT was the modality of treatment for the patient’s prostate cancer, which is a significant cause of disease and mortality among men. Each year, 1.6 million men are diagnosed with, and 366,000 men die from, prostate cancer [2]. The radio nucleotides used for prostate cancer most commonly are 125-Iodine and 102-Palladium [1].

BT seeds (pellets) are tiny, linear seeds with a silver or titanium shell that has a distinct radiopaque pattern that is easily identifiable on chest radiograph [3]. The common dimensions of the seeds are 0.8 mm in width and 4-5 mm in length and can pass through an 18-gauge needle when being implanted [4]. Due to the metallic shell, these seeds can be visualized on a radiograph. Conventional plain radiography is a reasonable modality to detect foreign bodies and to determine whether it is in a critical location. Radiography has 36% sensitivity and 100% specificity [5]. CT is standard for imaging and localizing foreign bodies because their shape and size are accurately reproduced. Ultrasonography might be useful for locating superficial foreign bodies [6] and may not provide value here. Scintigraphy is another modality that can be employed and has 100% sensitivity and 100% specificity [7].

The key parameters influencing the incidence and rate of seed embolization are the planning volume, number of seeds, seed placement, and type of seeds used (stranded vs. free). Planning volume determines the number of seeds required for implantation; thus, a higher planning volume requires a greater number of seeds, and this increases the risk of embolization. Seed placement is another important factor, with implantation being preferred in the periphery of the prostate to reduce radiation dose to the urethra. Stranded seeds are superior to free seeds, as they have a tissue-absorbable suture linking the seeds together with absorption occurring between 60 and 90 days postoperatively, which is adequate time for epithelialization to occur, thus decreasing the risk of embolization [8-10].

Seed migration is a rare complication, occurring in <1% of cases post-implantation, with pulmonary embolization occurring in up to 20% of these cases [11]. In a study by Stone et al., a total of 21,654 seeds were implanted, with post-implant chest X-rays obtained at a median of 912 days revealing at least one seed embolus in four patients (1.7%). Of the 21,654 seeds, 10 (0.005%) were found in the lungs [12]. Steinfeld et al. reported the first case of pulmonary seed embolization in 1991 [13]. The prostate has a well-developed venous plexus and seeds are commonly implanted around the periphery (as mentioned above). Thus, the seeds can enter blood vessels and are transported in the blood, in this case, to the lungs. The literature mentions other areas of embolization, including the bladder and heart.

A literature search doesn't mention the embolization of BT seeds when used for gynecologic cancers. However, Fan et al. studied seed migration following parotid gland interstitial brachytherapy in 321 patients. Six patients had migration to the lung, with an incidence of 1.87% and individual seed migration of 0.04% [14].

The differential diagnosis to consider for brachytherapy seed migration to the pulmonary arteries includes foreign objects, plate-like atelectasis, and emboli from iodized oil, acrylic cement, or metallic mercury [3].

The potential complications of embolization include iatrogenic irradiation, radiation pneumonitis, and the potential to induce carcinogenesis [3,9], particularly in the first few months after the procedure. The literature does not mention a role for routine chest radiographs post-implantation. In summary, it is essential to know that BT poses a minor risk of embolization and provides reassurance to the patient.

## Conclusions

The diagnosis of prostate BT embolization to the lung can be made with imaging techniques, such as chest radiograph followed by CT chest, once a thorough history and examination are carried out. At present, the literature does not mention a role for post-implantation chest radiographs. The patient needs to be reassured once this diagnosis is made.

## References

[1] Skowronek J (2017). Current status of brachytherapy in cancer treatment - short overview. J Contemp Brachytherapy.

[2] Pernar CH, Ebot EM, Wilson KM, Mucci LA (2018). The epidemiology of prostate cancer. Cold Spring Harb Perspect Med.

[3] Calvert AD, Dyer AW, Montgomery VA (2017). Embolization of prostatic brachytherapy seeds to pulmonary arteries: a case study. Radiol Case Rep.

[4] Aronowitz JN, Rivard MJ (2013). The phylogeny of permanent prostate brachytherapy. J Contemp Brachytherapy.

[5] Aras MH, Miloglu O, Barutcugil C, Kantarci M, Ozcan E, Harorli A (2010). Comparison of the sensitivity for detecting foreign bodies among conventional tomography and ultrasonography. Dentomaxillofac Radiol.

[6] Stockmann P, Vairaktaris E, Fenner M, Tudor C, Neukam FW, Nkenke E (2007). Conventional radiographs: are they still the standard in localization of projectiles?. Oral Surg Oral Med Oral Pathol Oral Radiol Endod.

[7] Kono Y, Kubota K, Mitsumoto T (2008). Scintigraphic detection of 125I seeds after permanent brachytherapy for prostate cancer. J Nucl Med.

[8] Eshleman JS, Davis BJ, Pisansky TM (2004). Radioactive seed migration to the chest after transperineal interstitial prostate brachytherapy: extraprostatic seed placement correlates with migration. Int J Radiat Oncol Biol Phys.

[9] Tapen EM, Blasko JC, Grimm PD (1998). Reduction of radioactive seed embolization to the lung following prostate brachytherapy. Int J Radiat Oncol Biol Phys.

[10] Merrick GS, Butler WM, Dorsey AT, Lief JH, Benson ML (2000). Seed fixity in the prostate/periprostatic region following brachytherapy. Int J Radiat Oncol Biol Phys.

[11] Kunos CA, Resnick MI, Kinsella TJ, Ellis RJ (2004). Migration of implanted free radioactive seeds for adenocarcinoma of the prostate using a Mick applicator. Brachytherapy.

[12] Stone NN, Stock RG (2005). Reduction of pulmonary migration of permanent interstitial sources in patients undergoing prostate brachytherapy. Urology.

[13] Steinfeld AD, Donahue BR, Plaine L (1991). Pulmonary embolization of iodine-125 seeds following prostate implantation. Urology.

[14] Fan Y, Huang M, Zhao Y (2017). Radioactive seed migration following parotid gland interstitial brachytherapy. Brachytherapy.

